# Scientific Items

**Published:** 1878-10-20

**Authors:** 


### *SCIENTIFIC ITEMS.*

### PHOTOGRAPHING WITH LIGHTNING.

[FROM THE SOUTHERN MEDICAL RECORD.]

Mr. A. J. Jarmin communicates to the *Photographic News* an account of experiments made to take negatives during a thunder storm. He sensitized a plate in the usual way and placed it at the back of a negative. Four flashes of lightning were counted, and, upon developing, the image came out as clear and quick as if taken by ordinary daylight. One flash with a weak negative gave a fair transparency. He next tried with the camera. After getting everything nicely in focus, through his studio window, which was done by the aid of lightning, he obtained a photograph with twenty flashes, the view being down a street. The experiment proves that the chemical process of ligntning is equal to the electric light produced artificially and nearly eaqual to daylight. Photographs of the electric spark itself have been tataken by aid of a Ruhmkorff coil. We are the fortunate possessor of several stereoscopic photograps of such Ruhmkorff spark flashes, which are very instructive to look at and study details, because while the spark itself lasts only a very small fraction of a second, the stereoscopic photograph of the same, being of course permanent, admits of careful and prolonged inspection.

### THE MOON.

A large crater or cavity not heretofore observed, has been discovered in the moon, giving evidence of violent activity, on its surface. Interesting phothographic views have also been taken, which when compared with a phothographic view of the face of Mount Vissuvius bears a resemblance so striking as to leave no doubt of a like volcanic condition, either extinct or active, upon the face of our satelite.

*An Electric Awakener.*—Mr. F. Peppard is the inventor of a curious contrivance for awakening a sleeper at any required hour The apparatus is to be fixed on an ordinary clock; it is so arranged that when the hour-hand of the clock touches a button an electric circuit is completed; the minute-hand passes over the button without effect. There are a series of holes for the different hours, into any one of which the button can be pushed, according to the time selected for awakening. The completion of the electric circuit may ring a bell, or sound any other of the ordinary methods of alarm. But this contrivance has a yet more effective method for arousing a deaf man or any sleeper who is willing beforehand to prepare himself for a shock. A bracelet is provided which can be put on the wrist at the same time of retireing; to this flexible wires are attached, and the electric discharge will pass through it at the appointed hour.---*Journal of Chemistry.*

### THE POLYMICROSCOPE.

A recent number of *Nature* states that "a new improvement in the microscope is reported from Germany. Herr I, von Lenhossek has constructed an apparatus which permits no less than sixty microscopical preparations being observed in immediate succession, without the trouble of changing the slides and readjustment of the object-glass. Its construction is similar in principle to that of the well-known revolving sterescopes, and the inventor has given the new apparatus the name of "polymicroscope."—*Pacific Med. and Sur. Jour.*

